# A blueprint for engaging communities to reduce inequities in maternal and child health: evidence from rural Guatemala

**DOI:** 10.1186/s12939-022-01753-x

**Published:** 2023-02-28

**Authors:** William T. Story, David F. Pyle

**Affiliations:** 1 Department of Community and Behavioral Health, University of Iowa College of Public Health, Iowa City, Iowa USA; 2 Independent Consultant, Portland, Oregon USA

**Keywords:** Maternal health, Child health, Community health, Primary health care, Community-based primary health care, Implementation research, Census-Based Impact-Oriented Approach, Care Groups, Community birthing centers, Guatemala, Equity, Curamericas Global, Curamericas/Guatemala

As we approach the 50th anniversary of the International Conference on Primary Health Care, which was held at Alma-Ata, and the resulting Declaration that promoted “Health for All,” significant progress has been made [1, 2]. Community engagement and participation have been at the forefront of achieving “Health for All” and reducing disparities in maternal and child health (MCH) outcomes [3–6]. Recently, there have been efforts to acknowledge community engagement as an important aspect of strengthening health systems [7], including the critical role of community health workers (CHWs) and women’s groups [8–10]. In low-resource settings, CHWs and women’s groups work together to empower their communities and address the most pressing health problems, especially those affecting the most vulnerable populations (i.e., children under five and pregnant and lactating mothers). These community structures serve as vital links to the existing health system, which is often centered at health posts and clinics. However, it is difficult to sustain active community participation in the health system unless community engagement is deeply embedded in the fabric of society. Furthermore, there are very few blueprints for how to sustainably engage communities to have a positive impact on health systems and health outcomes [3]. This supplement provides such a blueprint.

The non-governmental organizations (NGOs), Curamericas Global and its partner Curamericas/Guatemala, implemented a Maternal and Child Health Project (hereafter referred to as the Project) in isolated rural mountainous communities of the Western Highlands of the Department of Huehuetenango, Guatemala from 2011 to 2015 reaching a population of nearly 100,000 people. This 10-paper supplement provides a carefully documented account of not only *what* was achieved by the Project, but more importantly *how* the Project was carried out. The Curamericas team describes in detail the community-based strategies, the implementation research protocols and data collection procedures, the coverage of evidence-based MCH prevention and treatment interventions, the nutrition and mortality outcomes, the effect on women’s empowerment, and perspectives on the sustainability and cost-effectiveness of the overall strategy. These efforts not only improved equity in access to care and health outcomes among these marginalized Indigenous Maya families, but they also serve as a roadmap for other organizations working with marginalized and vulnerable populations in rural locations throughout the world.

This supplement focuses on the Census-Based, Impact-Oriented Plus (CBIO+) Approach, which is based on over 40 years of field work. As described by Valdez and colleagues in Paper 1 of this series [11], the CBIO+ Approach combines three evidence-based MCH strategies–the CBIO Approach, Care Groups, and Community Birthing Centers–to strengthen health systems and improve MCH in isolated, rural areas where individuals (especially women) are marginalized by oppressive social and political structures. First, the CBIO Approach was developed by Curamericas Global (then called Andean Rural Health Care) in the 1980s in rural Bolivia and achieved high coverage of MCH interventions as well as a reduction in child mortality by 50% [12–16]. CBIO consists of working in partnership with the community to develop a census, register all households, identify local public health priorities, develop a plan to address these priorities, and assess population health improvements over time [12, 13, 17]. Second, the Care Group Approach was developed by the NGO World Relief in the 1990s in Mozambique and also achieved significant improvements in household-level behavior change and nutrition as well as reductions in child mortality [18–22]. The Care Group Approach is a cascaded community empowerment strategy whereby one female Care Group Volunteer is assigned 10 to 15 households. Then, a Care Group Facilitator meets with groups of 5 to 12 Care Group Volunteers every two to four weeks to share one or two new educational messages, which the Volunteers then share with the mothers in their assigned households [21]. Curamericas successfully piloted a combination of the CBIO and Care Group approaches in Guatemala (2002–2007) and Liberia (2008–2013) [23, 24]. Third, the Community Birthing Center Approach (also known as *Casas Maternas*) is a community-engaged process whereby communities work together to construct, staff, and operate a local center specifically for childbirth. This center is staffed by an auxiliary nurse (with training in obstetrics) and respects the local customs of the population they are serving, including the important role of a traditional midwife who is welcome to join the team if a mother requests her assistance for her birth. Each birthing center includes a local emergency transport system and a local insurance system to help families pay for the high cost of transport to the referral hospital if needed [25]. These three approaches comprised the CBIO+ Project, which was designed to be a model for others to replicate, especially when remote, isolated, underserved populations needed access to health promotion and quality health care.

After the Project is described in detail in Paper 1 [11], the site, design, and methods are laid out by Perry and colleagues in Paper 2 [26]. The study area was divided into two groups of communities where Project interventions were implemented at different times: Area A (from 2011 to 2015) and Area B (from 2013 to 2015). Area B served as a quasi-comparison area for the first 2 years. After implementation started in Area B in 2013, this area was used to observe the potential dose-response relationships for each intervention as well. This paper describes the primary outcomes in detail as well as the methods for data collection. A variety of quantitative methods (e.g., household survey data, anthropometric data, and quality of clinical care data) and qualitative methods (e.g., focus group discussions, group interviews, key informant interviews, and verbal autopsies) were employed. This paper demonstrates meticulous attention to data collection used by the Project, which allowed for the robust analysis of multiple aspects of the CBIO+ Approach—from documenting implementation processes to estimating the number of lives saved by the Project.

In Paper 3, Blanco and colleagues [27] describe changes in 24 indicators of MCH knowledge, practice, and coverage (KPC) over time and between the two Project areas. Twenty-one indicators in Area A and 19 indicators in Area B showed significant improvements from baseline to endline. Furthermore, when the outcomes in Area A were compared with Area B, more favorable outcomes were achieved in Area A than in Area B, which is consistent with the hypothesized dose-response effect of the CBIO+ interventions. Although Area A out-performed Area B, the improvements in Area B were noteworthy because this demonstrates that the CBIO+ Approach can achieve significant increases in coverage in a relatively short period of time.

In Paper 4, Perry and colleagues [28] describe the nutrition-focused parts of the CBIO+ Approach (including health education, cooking demonstrations, and growth monitoring and counseling) and assess the effect of the interventions on childhood nutrition status (i.e., stunting, wasting, and underweight) from baseline to endline in both Project areas. This study showed that stunting declined from 74.5% to 39.5% in Area A; however, no improvements in nutritional status were observed in Area B. This result was even more impressive considering that there was no significant improvement in stunting in the rural regions of the Department of Huehuetenango (according to Demographic and Health Survey data) between 1999 and 2015. The improvements in Area A were likely due to improvements in dietary practices [27], improvements in childhood infection prevention and control [27], and the empowering effects of Care Groups on mothers [29]. Although the Project did not have an effect on wasting and underweight (due to disruptions in and the deterioration of the local and national health systems), the Project should be commended on its impact on stunting in a remote, Indigenous population.

Perry and colleagues continue their assessment of the CBIO+ Approach in Paper 5 by examining maternal, neonatal, and under-5 child mortality through two different approaches: (1) direct prospective measurement of mortality over time through the registration of births and deaths by Care Group Volunteers, and (2) indirect estimation by comparing the change in population coverage of evidence-based interventions shown to reduce mortality [30]. The vital events registration method revealed a 59.1% decline in maternal mortality from 632 deaths per 100,000 live births in Years 1 and 2 to 257 deaths per 100,000 live births in Years 3 and 4. However, this method revealed no significant declines in either neonatal or under-5 child mortality, which may be due to incomplete registration of neonatal and under-5 child deaths during the first 2 years of the Project. The indirect estimates based on changes in population coverage of evidence-based interventions revealed net declines of 12%, 5%, and 22% in maternal, neonatal, and under-5 mortality, respectively. It was also found that 44% of the under-5 deaths occurred during the first month of life and 62% of the neonatal deaths occurred on the first day of life. Furthermore, this study described the primary causes of maternal mortality (postpartum hemorrhage), neonatal mortality (birth asphyxia and intrapartum complications), and under-5 child mortality (pneumonia). Having a home birth was also associated with an eight-fold increased risk of both maternal and neonatal mortality. This study demonstrates that mortality assessments are feasible with minimal external technical support and can help guide decision-making for primary health care programs.

In Paper 6, Olivas and colleagues use a mixed-methods approach to demonstrate how Community Birthing Centers managed pregnancy complications [31]. To reduce the high burden of maternal mortality in this Indigenous population, the Project addressed the cultural insensitivity of the delivery process by eliminating linguistic, economic, and physical barriers. The study showed that well-trained and supported auxiliary nurses stationed at the Birthing Centers provided high quality, physically accessible, and culturally appropriate care. This resulted in the referral of complicated cases to facilities that could deal with their particular problems, thus avoiding unnecessary deaths and reducing maternal mortality. The most common birth complication was footling breech presentation, of which 90% were successfully delivered at Birthing Centers. The study ends with an excellent description of the decision-making process for Birth Center staff, and the integral role of the family when determining whether to resolve or refer a complication. Despite the success of the Community Birthing Center intervention, barriers to families accepting referrals (e.g., cost of transportation for referral) need to be addressed.

In Paper 7, Gregg and colleagues address an important gap in the literature about the Care Group Approach and its effectiveness, namely the empowering effect on the women who participate in the Care Groups [29]. This study describes how the Care Groups established by the Project increased the perceived social status, self-efficacy, decision-making autonomy, and formation of social capital among the women who were part of the Care Groups. Of particular importance was the empowerment of the marginalized Indigenous women in a male-dominated society. Not only does this suggest that there was a better chance of the population benefitting from the CBIO+ Approach, but it also means that it has an improved chance of being sustained by the local population. This study has implications for all projects that integrate the Care Group Approach into their efforts to improve health and health equity.

In a similar vein, Stollak and colleagues use a mixed-methods approach in Paper 8 to examine women’s empowerment, which hypothesized that the CBIO+ Approach would improve women’s decision-making autonomy as well as community participation [32]. This study also explored the barriers and facilitators of women’s empowerment. The Project provided women with knowledge and skills through the Care Group process that enabled them to take a more active role in their own and their family’s health decisions, and found that women significantly increased their participation in community meetings. Although these women still face barriers to their own agency, achieving such an improvement in participation and autonomy in a conservative cultural setting is a major accomplishment and is bound to have a positive impact on the health status of the women and their families in the future.

It was not just the beneficiary population that had a positive opinion of the Project and the CBIO+ Approach. In Paper 9, Lamden and colleagues describe how the key stakeholders (e.g., the Project staff and local government health providers) in the Project area also thought that the CBIO+ Approach was worthwhile and had a significant impact on the health and nutritional status of young children and mothers [33]. The consensus was that it would be beneficial if the CBIO+ Approach was integrated into the rural public health system. There was particularly strong support for the inclusion of community members in the program planning process; this was seen as a major improvement. Nonetheless, problems still arose, including internal community conflicts, coordination problems between the Project and the community, difficulties maintaining a high level of community participation, and overcoming male opposition to women’s participation in Care Groups. Some modifications to the CBIO+ Approach were suggested and considered, but, in the end, health care workers and the community were highly supportive and wanted the Project to continue.

In the final paper of the series (Paper 10), Perry and colleagues summarize the major findings from each of the first nine papers in the series, review the support for the original hypotheses, and conduct a cost-effectiveness analysis for the Project as a whole [34]. As you have read in this introduction, the Project achieved success in a number of areas, including (but not limited to) expanding coverage of and access to important MCH interventions, improving the nutritional status of children, reducing maternal and child deaths, improving the quality of care through Community Birthing Centers, and empowering women and strengthening social capital throughout the Project areas. Overall, the CBIO+ Approach was found to be a cost-effective way to reduce inequity in MCH outcomes, including mortality.

This series provides further evidence that the CBIO Approach (in combination with Care Groups and Community Birthing Centers), is an effective strategy for reaching marginalized populations in remote rural areas of the world. To continue to make progress towards “Health for All” and the Sustainable Development Goals for women and children, we must focus on health equity [35]. To make health promotion and health care access more equitable, there is a need to dismantle systems of oppression that continue to disadvantage certain groups of people, like the Indigenous Maya population in rural Guatemala. We agree with Perry and colleagues, who state in the last paper of this series that “by engaging communities, building trust and establishing partnerships from the outset of the program, CBIO+ can provide the necessary comprehensive approach to improve the health of populations in resource-limited settings [34].” With the renewed interest in strengthening health systems and investing in community health worker programs [7–9], the CBIO+ Approach is very relevant today. This series of papers provides a blueprint to improve health equity, especially in remote, rural locations, by engaging communities and sustaining participation in primary health care, developing systems for rigorous monitoring and evaluation, and, ultimately, breaking down oppressive systems so that all women and children can thrive.

## Data Availability

Not applicable.

## Abbreviations

CBIO: Census-Based, Impact-Oriented
CBIO+: The Expanded CBIO Approach (joint implementation of the CBIO Approach together with Care Groups and Community Birthing Centers called *Casas Maternas Rurales* as a single Project strategy)
CHW: Community health worker
KPC: Knowledge, practice, and coverage survey
MCH: Maternal and child health
NGO: Non-governmental organization
